# Author Correction: Cdo1-Camkk2-AMPK axis confers the protective effects of exercise against NAFLD in mice

**DOI:** 10.1038/s41467-026-75556-x

**Published:** 2026-07-14

**Authors:** Min Chen, Jie-Ying Zhu, Wang-Jing Mu, Hong-Yang Luo, Yang Li, Shan Li, Lin-Jing Yan, Ruo-Ying Li, Liang Guo

**Affiliations:** 1 School of Exercise and Health and Collaborative Innovation Center for Sports and Public Health, Shanghai University of Sport, Shanghai, 200438 China; 2 Shanghai Frontiers Science Research Base of Exercise and Metabolic Health, Shanghai University of Sport, Shanghai, 200438 China; 3 Key Laboratory of Exercise and Health Sciences of the Ministry of Education, Shanghai University of Sport, Shanghai, 200438 China

Correction to: *Nature Communications* 10.1038/s41467-023-44242-7; Article published online 18 December 2023

In the version of this article initially published, the Source Data files associated with Fig. 1d, 1l, 1o and Fig. 7h contained incorrect datasets owing to copy/paste errors during preparation. The published Fig. 1d, 1l and 1o were generated from the correct original data and are unaffected. Figure 7h has been replaced with the version generated from the correct original data, and the corresponding Source Data has been updated.

These corrections do not affect the statistical analyses, results, interpretation or conclusions of the article. The original data has been verified, and the HTML and PDF versions have been corrected. For comparison, the original Fig. 7 is available as Supplementary Information accompanying this amendment.

## Supplementary information

Original Fig. 7

