# Supplementary material for: Cdo1-Camkk2-AMPK axis confers the protective effects of exercise against NAFLD in mice
Source: Nat Commun. 2023 Dec 18;14:8391. doi: 10.1038/s41467-023-44242-7 (PMC10728194; doi:10.1038/s41467-023-44242-7)
Supplement: Supplementary file 1 — Supplementary information [file 41467_2023_44242_MOESM1_ESM.pdf]

Supplementary information for

# Cdo1-Camkk2-AMPK axis confers the protective effects of exercise against NAFLD in mice

Min Chen[1,2,3], Jie-Ying Zhu[1,2,3], Wang-Jing Mu[1,2,3], Hong-Yang Luo[1,2,3], Yang Li[1,2,3], Shan Li[1,2,3], Lin-Jing Yan[1,2,3], Ruo-Ying Li[1,2,3], Liang Guo[1,2,3*]

[1]School of Exercise and Health and Collaborative Innovation Center for Sports and Public Health, Shanghai University of Sport, Shanghai, China, 200438.
[2]Shanghai Frontiers Science Research Base of Exercise and Metabolic Health, Shanghai University of Sport, Shanghai, China, 200438.
[3]Key Laboratory of Exercise and Health Sciences of the Ministry of Education, Shanghai University of Sport, Shanghai, China, 200438.

*To whom correspondence should be addressed:
Liang Guo, E-mail: guoliang@sus.edu.cn
Professor of School of Exercise and Health, Shanghai University of Sport, Shanghai 200438, PR China.

This PDF file includes:
Supplementary Fig. 1 to 9
Supplementary Table 1

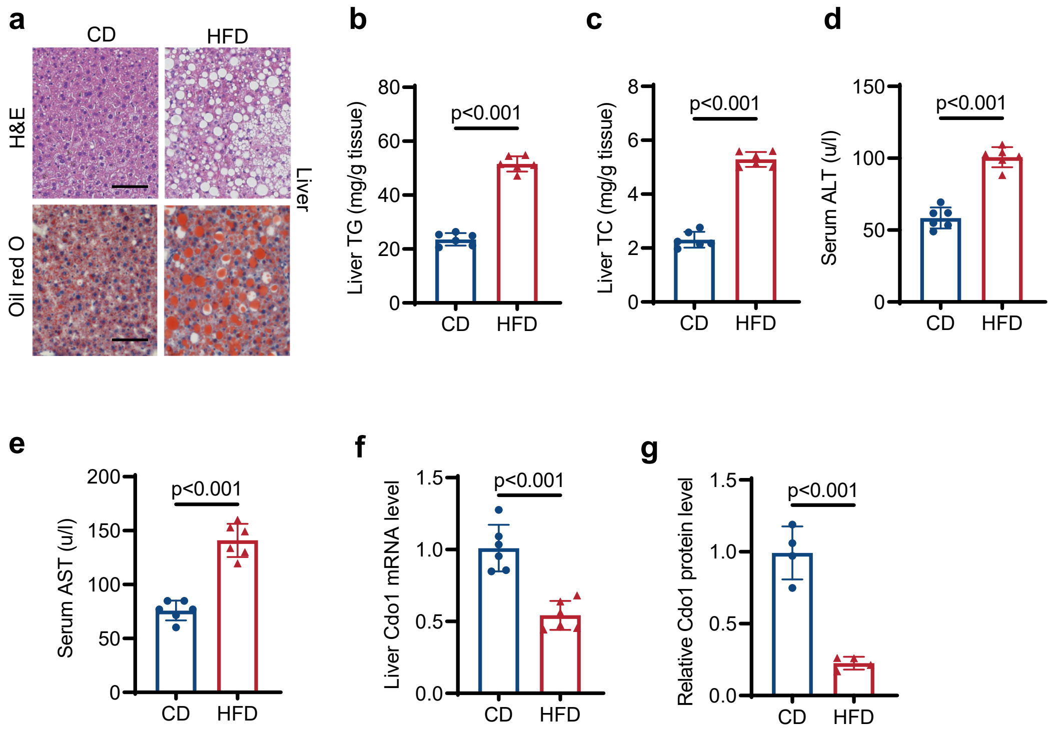


**Supplementary Fig.1 High-fat diet (HFD) induces hepatic steatosis in mice.** 6-week-old male mice were fed with chow diet (CD) or high-fat diet (HFD)-fed for 16 weeks before being sacrificed. **a** Representative images of hematoxylin and eosin (H&E) staining and Oil Red O staining analysis of liver sections. Experiments were performed 3 times and similar results were obtained. Scale bars, 50 μm. **b**, **c** Triglyceride (TG) and Cholesterol (TC) levels in mice livers, respectively (n=6 mice per group). **d** and **e** Serum alanine aminotransferase (ALT) and serum aspartate aminotransferase (AST) levels in mice, respectively (n=6 mice per group). **f** The Cdo1 mRNA level was determined in mice livers (n=6 mice per group). **g** Quantification of western blotting results of **Fig. 2c** (n=3 mice per group). For statistical analysis, unpaired two-tailed t tests were performed in **b-g**. All data show the means ± SD. Source data are provided as a Source Data file.

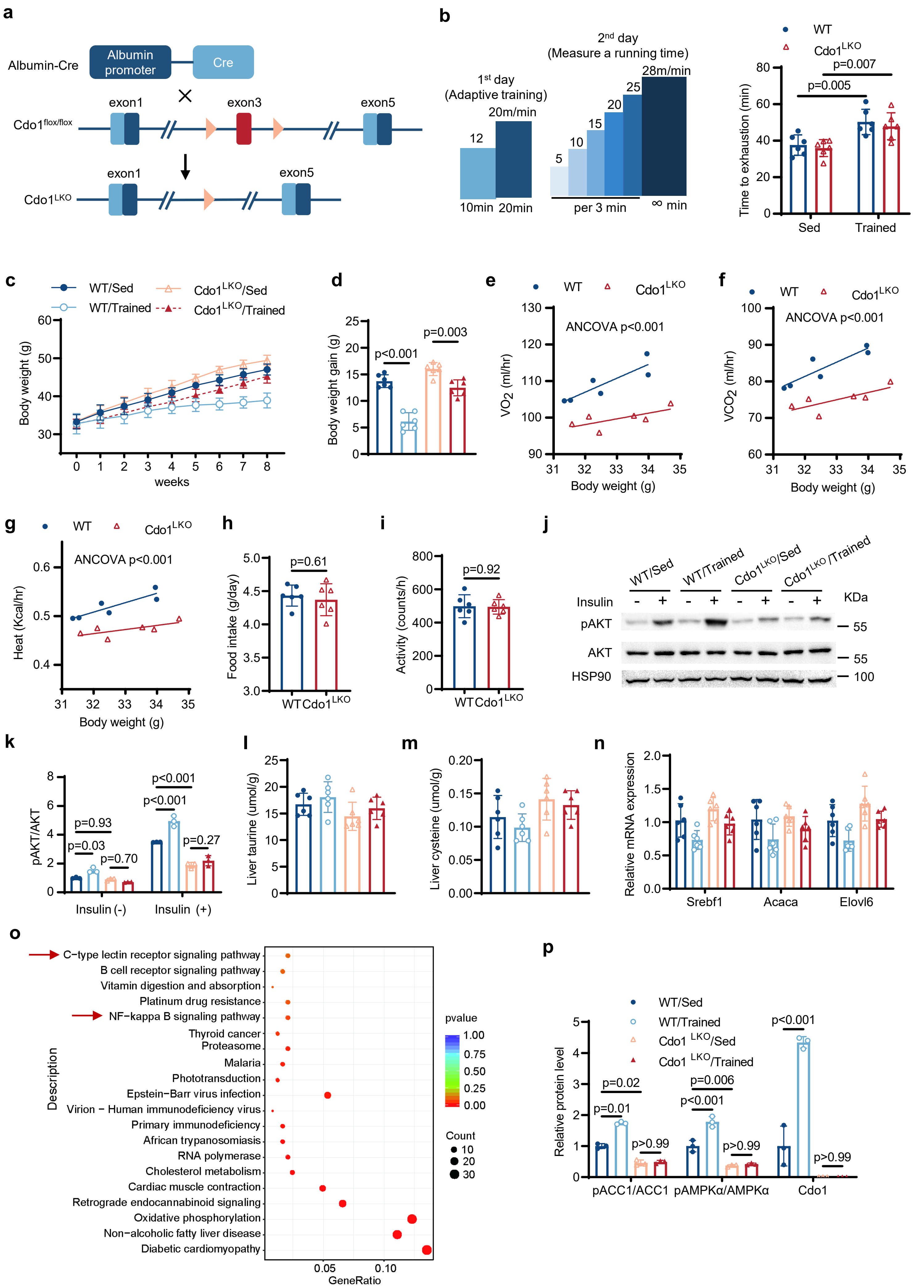
a
Albumin-Cre
Albumin promoter
Cre
Cdo1flox/flox
exon1
exon3
exon5
Cdo1LKO
exon1
exon5
b
1st day (Adaptive training)
12
20m/min
10min 20min
2nd day (Measure a running time)
5
10
15
20
25
28m/min
per 3 min
∞ min
WT
Cdo1LKO
Time to exhaustion (min)
p=0.005
p=0.007
Sed
Trained
c
WT/Sed
WT/Trained
Cdo1LKO/Sed
Cdo1LKO/Trained
Body weight (g)
weeks
d
Body weight gain (g)
p<0.001
p=0.003
e
WT
Cdo1LKO
ANCOVA p<0.001
VO2 (ml/hr)
Body weight (g)
f
WT
Cdo1LKO
ANCOVA p<0.001
VCO2 (ml/hr)
Body weight (g)
g
WT
Cdo1LKO
ANCOVA p<0.001
Heat (Kcal/hr)
Body weight (g)
h
Food intake (g/day)
p=0.61
WT Cdo1LKO
i
Activity (counts/h)
p=0.92
WT Cdo1LKO
j
WT/Sed
WT/Trained
Cdo1LKO/Sed
Cdo1LKO /Trained
Insulin
KDa
pAKT
AKT
HSP90
55
55
100
k
pAKT/AKT
p=0.93
p=0.03
p=0.70
p<0.001
p<0.001
p=0.27
Insulin (-)
Insulin (+)
l
Liver taurine (umol/g)
m
Liver cysteine (umol/g)
n
Relative mRNA expression
Srebf1
Acaca
Elovl6
o
C−type lectin receptor signaling pathway
B cell receptor signaling pathway
Vitamin digestion and absorption
Platinum drug resistance
NF−kappa B signaling pathway
Thyroid cancer
Proteasome
Malaria
Phototransduction
Epstein−Barr virus infection
Virion − Human immunodeficiency virus
Primary immunodeficiency
African trypanosomiasis
RNA polymerase
Cholesterol metabolism
Cardiac muscle contraction
Retrograde endocannabinoid signaling
Oxidative phosphorylation
Non−alcoholic fatty liver disease
Diabetic cardiomyopathy
Description
GeneRatio
pvalue
Count
p
WT/Sed
WT/Trained
Cdo1 LKO/Sed
Cdo1 LKO/Trained
Relative protein level
p=0.02
p=0.01
p>0.99
p=0.006
p<0.001
p>0.99
p<0.001
p>0.99
pACC1/ACC1
pAMPKα/AMPKα
Cdo1

**Supplementary Fig.2 Exercise-mediated alleviation of fatty liver in mice is blunted by hepatocyte-specific knockout of Cdo1 (Cdo1$^{LKO}$). a** Cdo1$^{LKO}$ mice (Cdo1$^{flox/folx}$/Albumin- Cre$^{+}$) were generated by crossbreeding Cdo1$^{flox/flox}$ and albumin promoter driven Cre transgenic mice. Cdo1$^{flox/flox}$ /Albumin-Cre$^{-}$ mice were used as wild-type control (WT). **b** Protocol for the treadmill exhaustion time test. Mice were treated as described in **Fig. 1a. c, d** Mice were treated as in **Fig. 2d** (n=6 mice per group). **c** The body weight changes of the mice during the 8 weeks of exercise. **d** The body weight gain of the mice for the 8 weeks of exercise. **e-i** Metabolic chamber measurements of the untrained mice (Sed) after 6 weeks of HFD feeding (n=6 mice per group). **e-g** Regression based analysis of $VO_2$, $VCO_2$ and heat production against body weight. **h** Food intake of the mice. **i** Physical activity of the mice. **j** WT and Cdo1$^{LKO}$ mice were treated as in **Fig. 2d**. After 14 weeks of HFD feeding (6 weeks of exercise training or not), mice were administered with insulin for 20min, after which they were sacrificed and liver tissues were harvested for analysis. Representative western blot analysis for AKT and its phosphorylation form (p-AKT, s473) in liver. HSP90α/β serves as an internal control (n=3 mice per group, and blots were performed 3 times and similar results were obtained). **k** Quantification of western blotting results of **j**. **l** to **n**, mice were treated as in **Fig. 2d** (n=6 mice per group). **l, m** Taurine and cysteine levels in mice livers, respectively. **n** The *Srebf1*, *Acaca* and *Elovl6* mRNA levels in mice livers. **o** Kyoto Encyclopedia of Genes and Genomes (KEGG) pathway analysis of upregulated genes based on RNA-seq data in **Fig. 3a**. **p** Quantification of western blotting results of **Fig. 3g**. Two-sided Wald tests without adjustment were performed in a **o**. Unpaired two-tailed t tests were performed in **h-i**; two-way analysis of variance plus Tukey's post hoc tests were performed in **b**, **d**, **k-n** and **p**; two-sided ANCOVA analyses were performed in **e-g**. All data show the means ± SD. Source data are provided as a Source Data file.

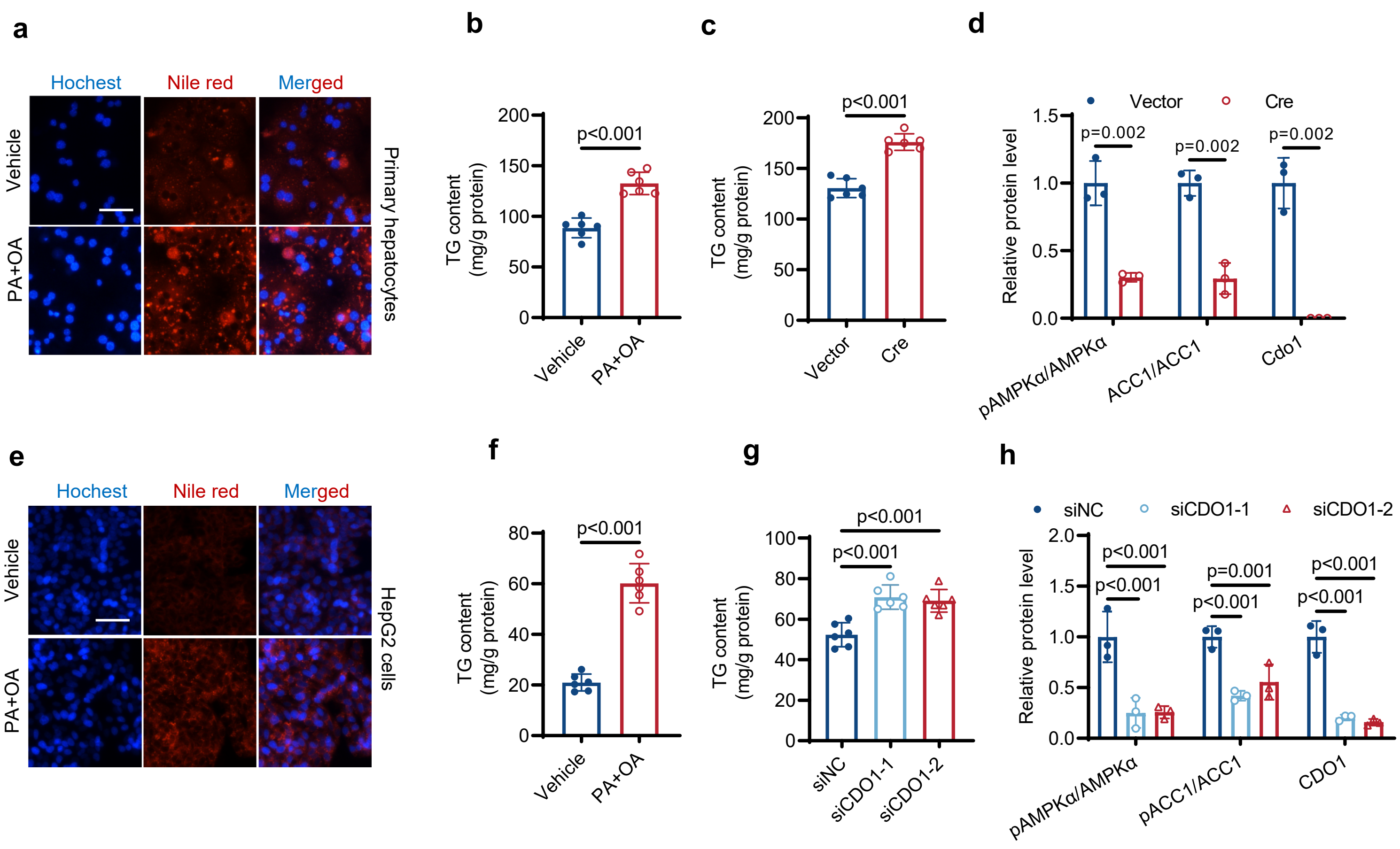


**Supplementary Fig.3 Cdo1 deficiency promotes steatosis in hepatocytes. a**, **b** Primary hepatocytes were treated with a mixture of 0.6 mM oleate and palmitate (FFAs) at a final ratio of 2:1 for 24 h. Then cells were harvested and analyzed. **a** Nile red and Hoechst staining of the cells. Scale bars, 50 μm. OA + PA, 0.6 mM oleate and palmitate. **b** Triglyceride (TG) levels in primary hepatocytes (n=6 independent biological replicates). **c, d** Cdo1$^{flox/flox}$ loci-containing primary hepatocytes were infected with adenovirus harboring Cre (AD-Cre) for the ablation of Cdo1. After 24 h, cells were treated with FFAs for 24 h. Then cells were harvested and analyzed (n=6 independent biological replicates). **c** TG levels in primary hepatocytes. **d** Quantification of western blotting results of **Fig. 4b**. **e, f** HepG2 cells were treated with a mixture of FFAs for 24 h. Then cells were harvested and analyzed. **e** Nile red and Hoechst staining of the cells. Scale bars, 50 μm. **f** TG levels in HepG2 cells (n=6 independent biological replicates). **g**, **h** HepG2 cells were transfected with the indicated small interfering RNA (siRNA). After 24 h, cells were treated with a mixture of FFAs for 24 h. Then cells were harvested and analyzed (n=6 independent biological replicates). **g** TG levels in HepG2 cells. **h** Quantification of western blotting results of **Fig. 4i**. Experiments were performed 3 times and similar results were obtained in **a** and **e**. Unpaired two-tailed t tests were performed in **b-d** and **f**; one-way analysis of variance plus Tukey's post hoc tests were performed in **g**; two-way analysis of variance plus Tukey's post hoc tests were performed in **h**. All data show the means ± SD. Source data are provided as a Source Data file.

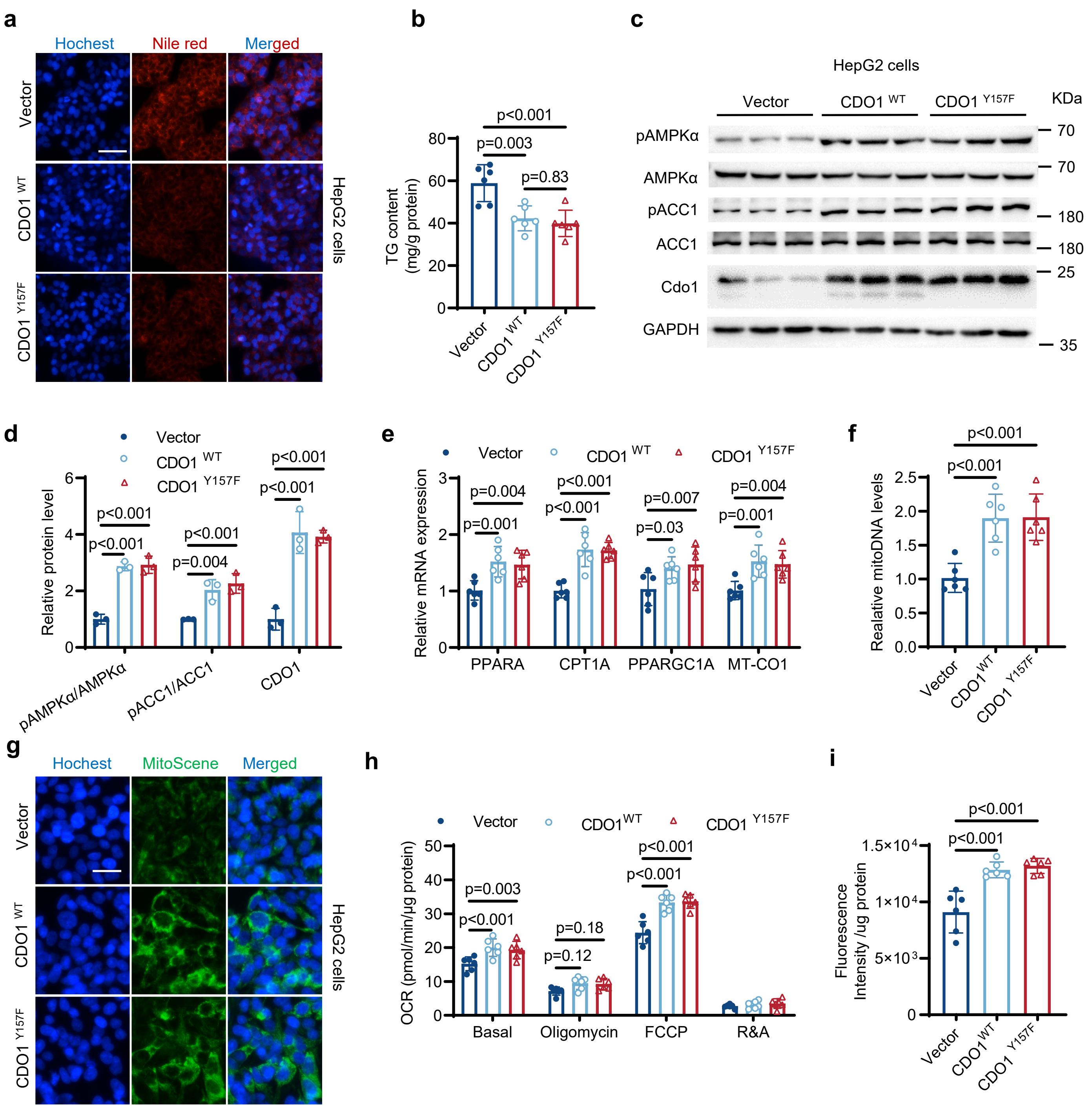


**Supplementary Fig.4 Overexpression of Cdo1 in HepG2 cells ameliorates hepatic steatosis.** HepG2 cells were infected with AD-Cdo1$^{WT}$, AD-Cdo1$^{Y157F}$ or Vector. After 24 h, cells were treated with a mixture of oleate and palmitate (FFAs) for 24 h. Then cells were harvested and analyzed. n=3 independent biological replicates for western blotting. n=6 independent biological replicates for other experiments. **a** Nile red and Hoechst staining of the cells. Scale bars, 50 μm. **b** Triglyceride (TG) levels in HepG2 cells. **c** HepG2 cells lysates were analyzed by western blotting. **d** Quantification of western blotting results of **c**. **e** The mRNA levels of the indicated genes were determined. **f** The mitochondrial DNA (mitoDNA) levels in HepG2. **g** MitoScene and Hoechst staining of the cells. Scale bars, 25 μm. **h** Respiration in HepG2 cells. OCR, oxygen consumption rate. FCCP, mitochondrial uncoupler. R&A, rotenone and antimycin A. **i** Fatty acid oxidation (FAO) levels in HepG2. Experiments were performed 3 times and similar results were obtained in **a, c** and **g.** One-way analysis of variance plus Tukey's post hoc tests were performed in **b**, **f** and **i**; two-way analysis of variance plus Tukey's post hoc tests were performed in **d**, **e** and **h**. All data show the means ± SD. Source data are provided as a Source Data file.

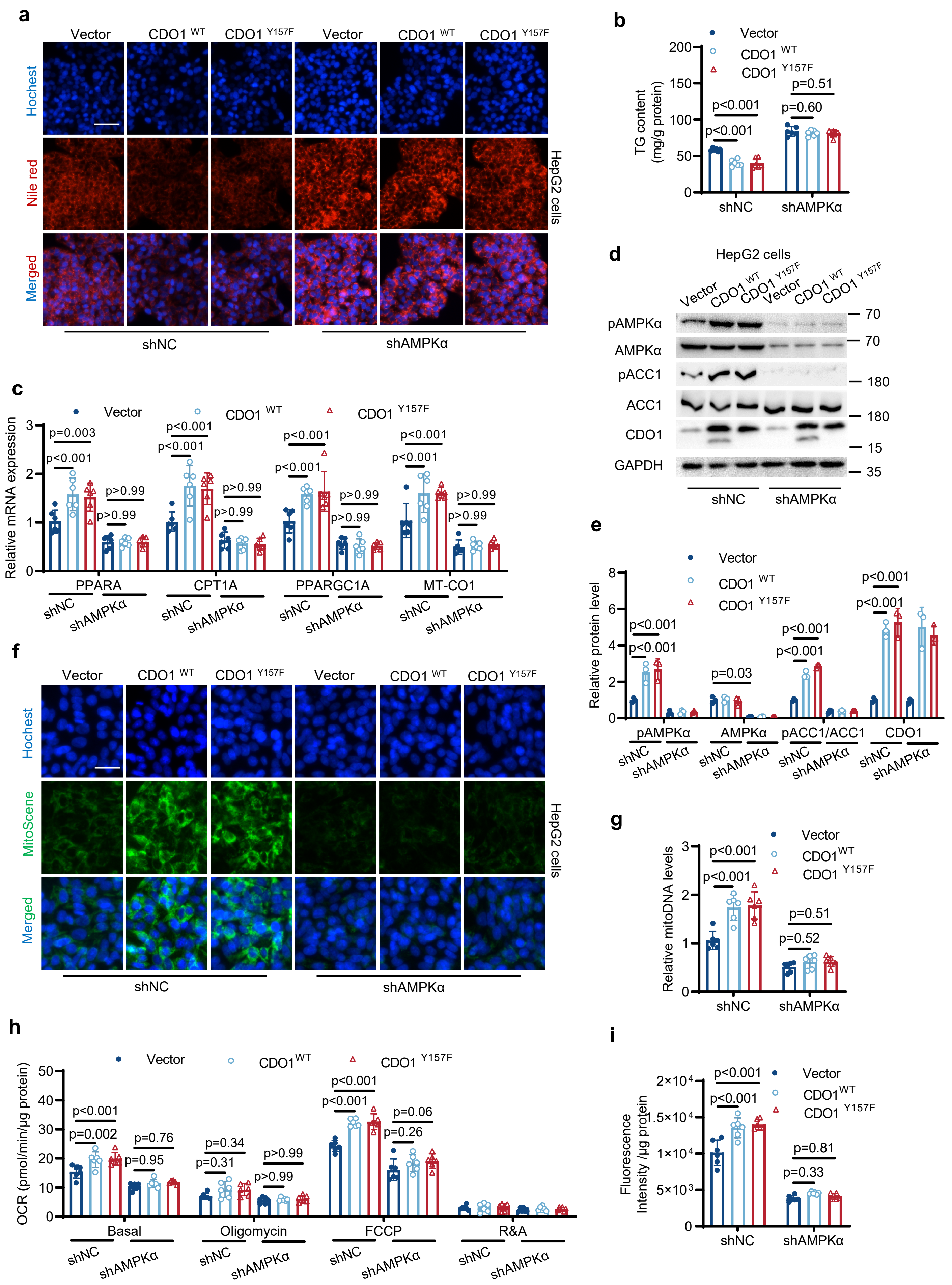

a
Vector
CDO1 WT
CDO1 Y157F
Hochest
Nile red
Merged
HepG2 cells
shNC
shAMPKα
b
Vector
CDO1 WT
CDO1 Y157F
TG content (mg/g protein)
p<0.001
p<0.001
p=0.51
p=0.60
shNC
shAMPKα
c
Relative mRNA expression
p=0.003
p<0.001
p>0.99
p>0.99
PPARA
CPT1A
PPARGC1A
MT-CO1
d
HepG2 cells
pAMPKα
AMPKα
pACC1
ACC1
CDO1
GAPDH
70
180
15
35
e
Relative protein level
p=0.03
pAMPKα
AMPKα
pACC1/ACC1
CDO1
f
MitoScene
g
Relative mitoDNA levels
p=0.52
h
OCR (pmol/min/μg protein)
p=0.002
p=0.76
p=0.95
p=0.34
p=0.31
p=0.06
p=0.26
Basal
Oligomycin
FCCP
R&A
i
Fluorescence Intensity /μg protein
p=0.81
p=0.33

**Supplementary Fig.5 AMPK signaling is required for Cdo1-mediated amelioration of steatosis in HepG2 cells.** HepG2 cells were infected with AD-Cdo1$^{WT}$, AD-Cdo1$^{Y157F}$ or Vector, together with the infection of adenoviruses harboring the indicated short hairpin RNA (shRNAs). After 24 h, cells were treated with a mixture of oleate and palmitate (FFAs) for 24 h. Then cells were harvested and analyzed. n=3 independent biological replicates for western blotting. n=6 independent biological replicates for other experiments. **a** Nile red and Hoechst staining of the cells. Scale bars, 50 μm. **b** Triglyceride (TG) levels in HepG2 cells. **c** The mRNA levels of the indicated genes. **d** HepG2 Cell lysates were analyzed by western blotting (n=3 independent biological replicates, and representative blot was shown). **e** Quantification of western blotting results of **d**. **f** MitoScene and Hoechst staining of the cells. Scale bars, 25 μm. **g** The mitochondrial DNA (mitoDNA) levels in HepG2. **h** Respiration in HepG2 cells. OCR, oxygen consumption rate. FCCP, mitochondrial uncoupler. R&A, rotenone and antimycin A. **i** Fatty acid oxidation (FAO) levels in HepG2. Experiments were performed 3 times and similar results were obtained in **a, d** and **f**. Two-way analysis of variance plus Tukey's post hoc tests were performed in **b**, **c**, **e**, and **g-i**. All data show the means ± SD. Source data are provided as a Source Data file.

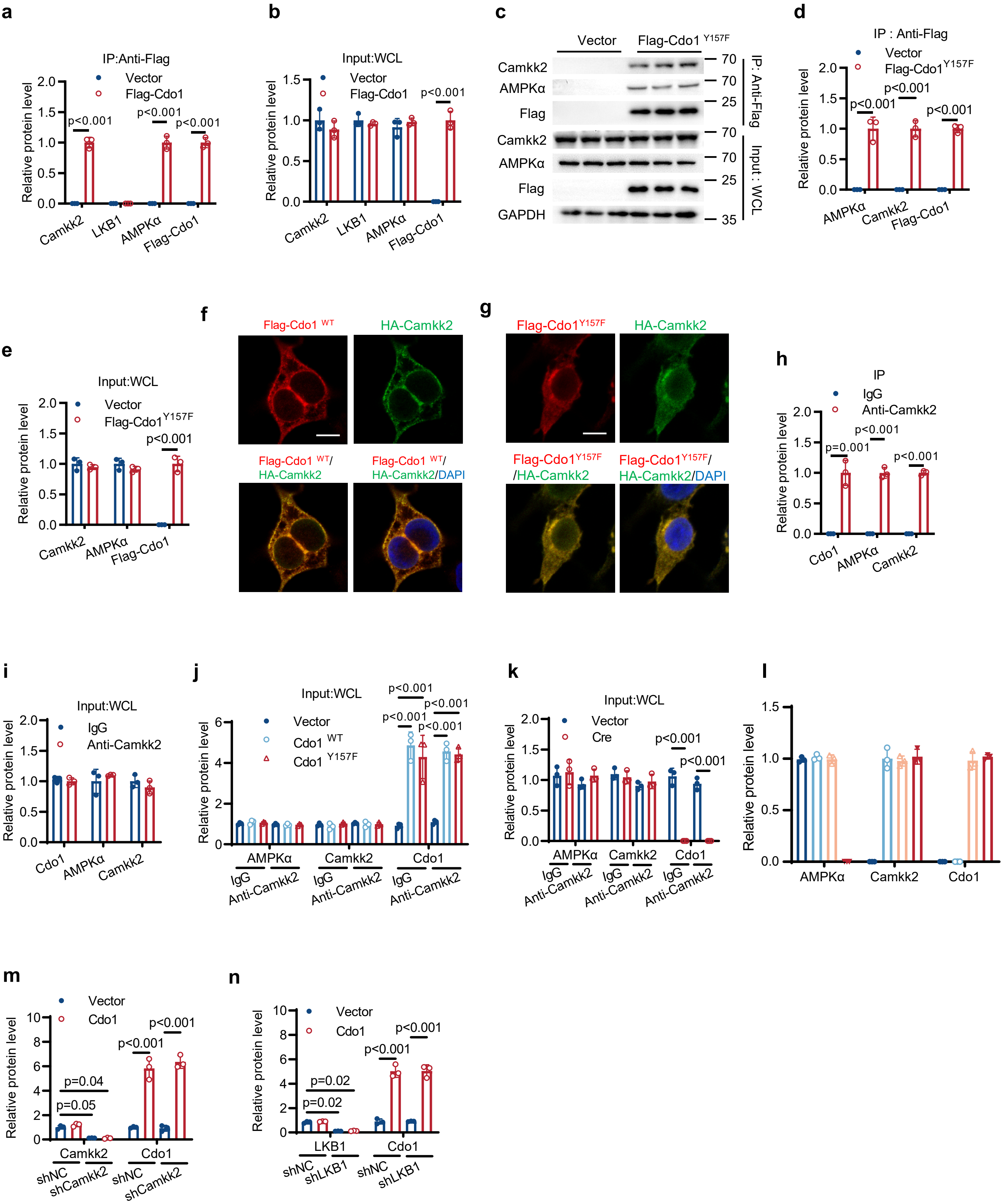
a
IP:Anti-Flag
Vector
Flag-Cdo1
Relative protein level
p<0.001
Camkk2
LKB1
AMPKα
Flag-Cdo1
b
Input:WCL
Vector
Flag-Cdo1
c
Vector
Flag-Cdo1 Y157F
Camkk2
AMPKα
Flag
GAPDH
IP: Anti-Flag
Input : WCL
d
IP : Anti-Flag
Flag-Cdo1Y157F
e
Input:WCL
f
Flag-Cdo1 WT
HA-Camkk2
Flag-Cdo1 WT/HA-Camkk2
Flag-Cdo1 WT/HA-Camkk2/DAPI
g
Flag-Cdo1Y157F
Flag-Cdo1Y157F/HA-Camkk2
Flag-Cdo1Y157F/HA-Camkk2/DAPI
h
IP
IgG
Anti-Camkk2
p=0.001
Cdo1
i
Input:WCL
j
Cdo1 WT
Cdo1 Y157F
k
Cre
l
m
Cdo1
p=0.04
p=0.05
shNC
shCamkk2
n
p=0.02
shLKB1

**Supplementary Fig.6 Cdo1 tethers Camkk2 and AMPKα to promote Camkk2-mediated AMPK phosphrylation.** **a**, **b** Quantification of Immunoprecipitation (IP) and Input western blotting results of **Fig. 7a** (n=3 independent biological replicates). **c** Primary hepatocytes were infected with AD-Flag-Cdo1$^{Y157F}$, and immunoprecipitation was performed using anti-Flag beads and followed by western blotting (n=3 independent biological replicates). WCL, whole cell lysate. **d** and **e** Quantification of IP and Input western blotting results of **c**. **f** and **g** HEK293T cells were co-transfected with plasmids encoding Flag-Cdo1$^{WT}$ or Flag-Cdo1$^{Y157F}$ and HA-Camkk2, followed by confocal analyses. Scale bars, 10 μm. **h**, **i** Quantification of IP and Input results of **Fig. 7d**. **j** Quantification of Input results of **Fig. 7e**. **k** Quantification of Input results of **Fig. 7g**. **l** Quantification of western blotting results of **Fig. 7i**. **m** Quantification of western blotting results of **Fig. 7k**. **n** Quantification of western blotting results of **Fig. 7m**. Experiments were performed 3 times and similar results were obtained in **c, f** and **g**. Unpaired two-tailed t tests were performed in **a**, **b**, **d**, **e**, **h** and **i**; two-way analysis of variance plus Tukey's post hoc tests were performed in **j-n**. All data show the means ± SD. Source data are provided as a Source Data file.

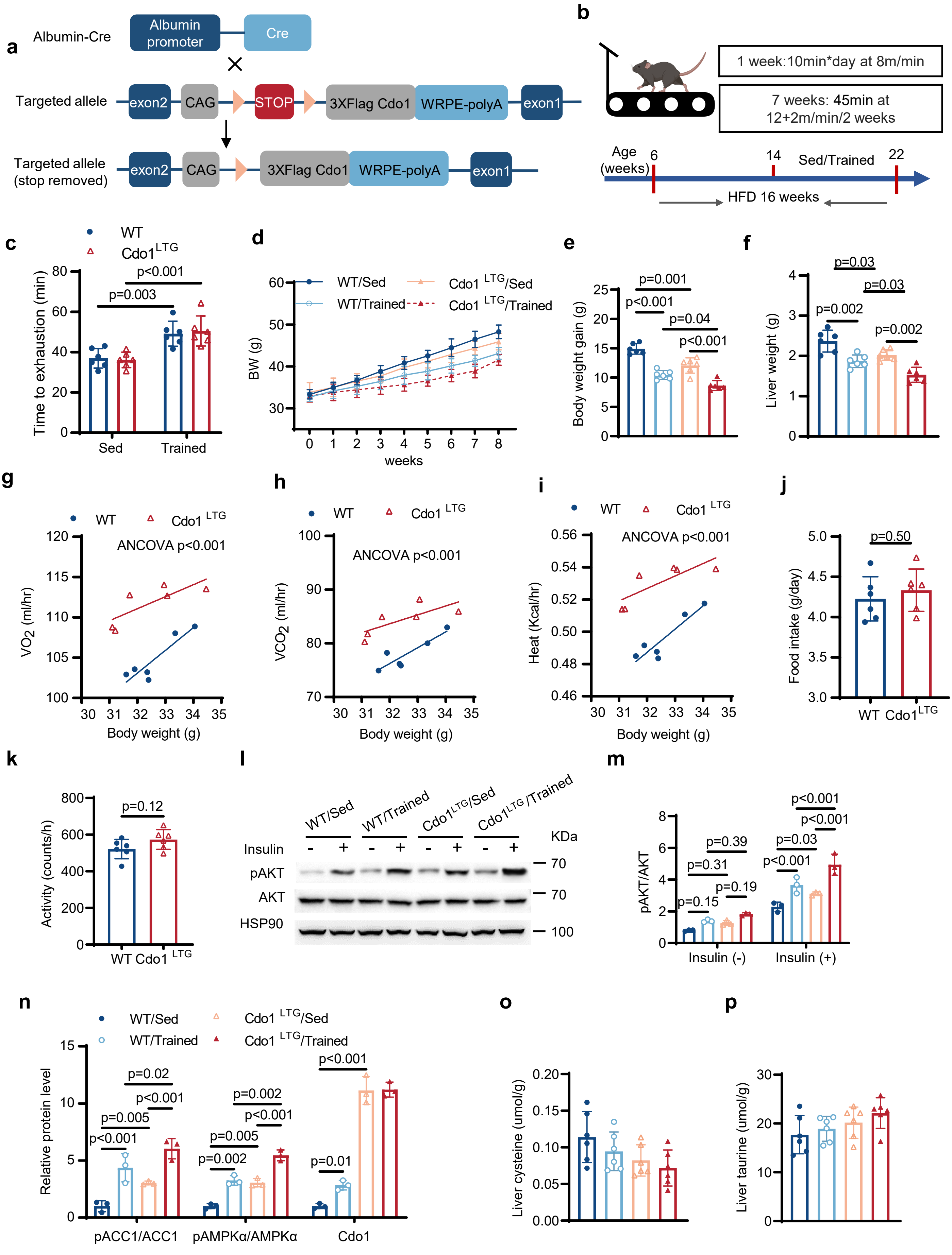

a
Albumin-Cre
Albumin promoter
Cre
Targeted allele
exon2
CAG
STOP
3XFlag Cdo1
WRPE-polyA
exon1
Targeted allele (stop removed)
b
1 week:10min*day at 8m/min
7 weeks: 45min at 12+2m/min/2 weeks
Age (weeks)
6
14
Sed/Trained
22
HFD 16 weeks
c
WT
Cdo1LTG
Time to exhaustion (min)
p=0.003
p<0.001
Sed
Trained
d
WT/Sed
WT/Trained
Cdo1 LTG/Sed
Cdo1 LTG/Trained
BW (g)
weeks
e
Body weight gain (g)
p=0.001
p<0.001
p=0.04
p<0.001
f
Liver weight (g)
p=0.03
p=0.03
p=0.002
p=0.002
g
WT
Cdo1 LTG
ANCOVA p<0.001
VO2 (ml/hr)
Body weight (g)
h
ANCOVA p<0.001
VCO2 (ml/hr)
Body weight (g)
i
ANCOVA p<0.001
Heat (Kcal/hr)
Body weight (g)
j
Food intake (g/day)
p=0.50
WT
Cdo1LTG
k
Activity (counts/h)
p=0.12
WT Cdo1 LTG
l
WT/Sed
WT/Trained
Cdo1LTG/Sed
Cdo1LTG /Trained
Insulin
pAKT
AKT
HSP90
KDa
70
70
100
m
pAKT/AKT
p<0.001
p<0.001
p=0.39
p=0.03
p=0.31
p<0.001
p=0.19
p=0.15
Insulin (-)
Insulin (+)
n
WT/Sed
WT/Trained
Cdo1 LTG/Sed
Cdo1 LTG/Trained
Relative protein level
p=0.02
p<0.001
p=0.005
p<0.001
p=0.002
p<0.001
p=0.005
p=0.002
p<0.001
p=0.01
pACC1/ACC1
pAMPKα/AMPKα
Cdo1
o
Liver cysteine (umol/g)
p
Liver taurine (umol/g)

**Supplementary Fig.7 Hepatocyte-specific overexpression of Cdo1 (Cdo1$^{LTG}$) and exercise cooperate to alleviate HFD-induced NAFLD in mice. a** Schema of liver specific Cdo1 transgenic (Cdo1$^{LTG}$) mouse model. **b** 6-week-old WT and Cdo1$^{LTG}$ mice were fed HFD for 16 weeks, with or without exercise in the last 8 weeks. The intervention program is illustrated. The representing mouse model was created using BioRender.com. **c** The time to exhaustion of Cdo1$^{LTG}$ and WT mice was measured by using the method as described in **Supplementary Fig. 2b**. Mice were treated as described in **Fig. 1a. d**-**f** Mice were treated as described in **b** before being sacrificed for analysis (n=6 mice per group). **d** The body weight changes of the mice during the 8 weeks of treadmill training. **e** The body weight gain of the mice for the 8 weeks of treadmill training. **f** Liver weights in mice. **g** to **k**, Metabolic chamber measurements of the untrained mice (Sed) after 6 weeks of HFD feeding (n=6 mice per group). **g-i** Regression based analysis of $VO_2$, $VCO_2$ and heat production against body weight of the mice. **j** Food intake of the mice. **k** Physical activity of the mice. **l** WT and Cdo1$^{LKO}$ mice were treated as described in **b**. After 14 weeks of HFD feeding (6 weeks of exercise training or not), mice were administered with insulin for 20min, after which they were sacrificed and the liver tissues were harvested for analysis. Representative western blot analysis for AKT and its phosphorylation form (p-AKT, s473) in liver (n=3 mice per group, and blots were performed 3 times and similar results were obtained.). **m** Quantification of western blotting results of **l**. **n** Quantification of western blotting results of **Fig. 8j**. **o, p** Mice were treated as described in **d**-**f**. Taurine and cysteine levels in mice livers. Unpaired two-tailed t tests were performed in **j-k**; two-way analysis of variance plus Tukey's post hoc tests were performed in **c**, **e**, **f** and **m-p**; two-sided ANCOVA analyses were performed in **g-i**. All data show the means ± SD. Source data are provided as a Source Data file.

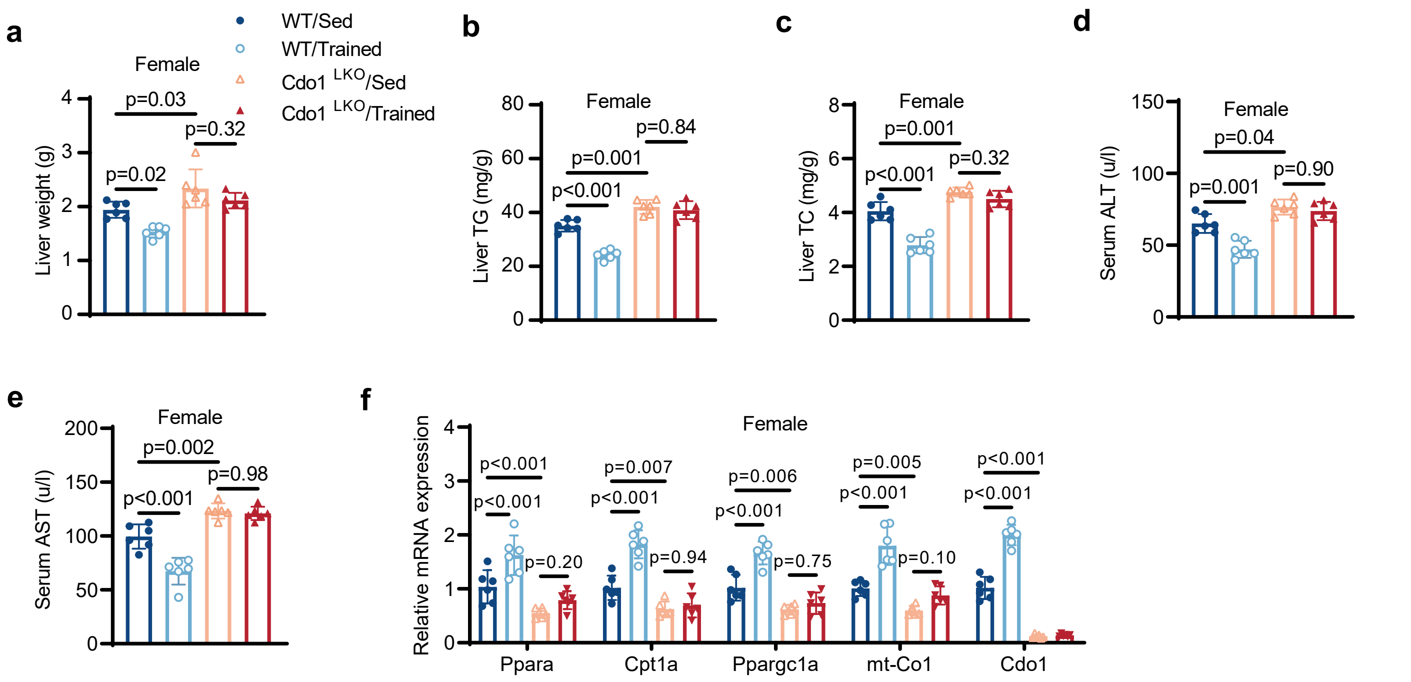


**Supplementary Fig.8 Exercise-mediated alleviation of fatty liver in female mice is blunted by Cdo1$^{LKO}$.** 6-week-old Cdo1$^{flox/flox}$ (WT) and Cdo1$^{LKO}$ female mice were fed HFD for 16 weeks, with or without exercise in the last 8 weeks. The intervention program is illustrated in **Fig. 2d**. Then mice were sacrificed for analysis (n=6 mice per group). **a** Liver weights in mice. **b**, **c** Triglyceride (TG) and Cholesterol (TC) levels in mice livers, respectively. **d**, **e** Serum alanine aminotransferase (ALT) and serum aspartate aminotransferase (AST) levels in mice, respectively. **f** The mRNA levels of the indicated genes were determined in mice liver. Two-way analysis of variance plus Tukey's post hoc tests were performed in **a-f**. All data show the means ± SD. Source data are provided as a Source Data file.

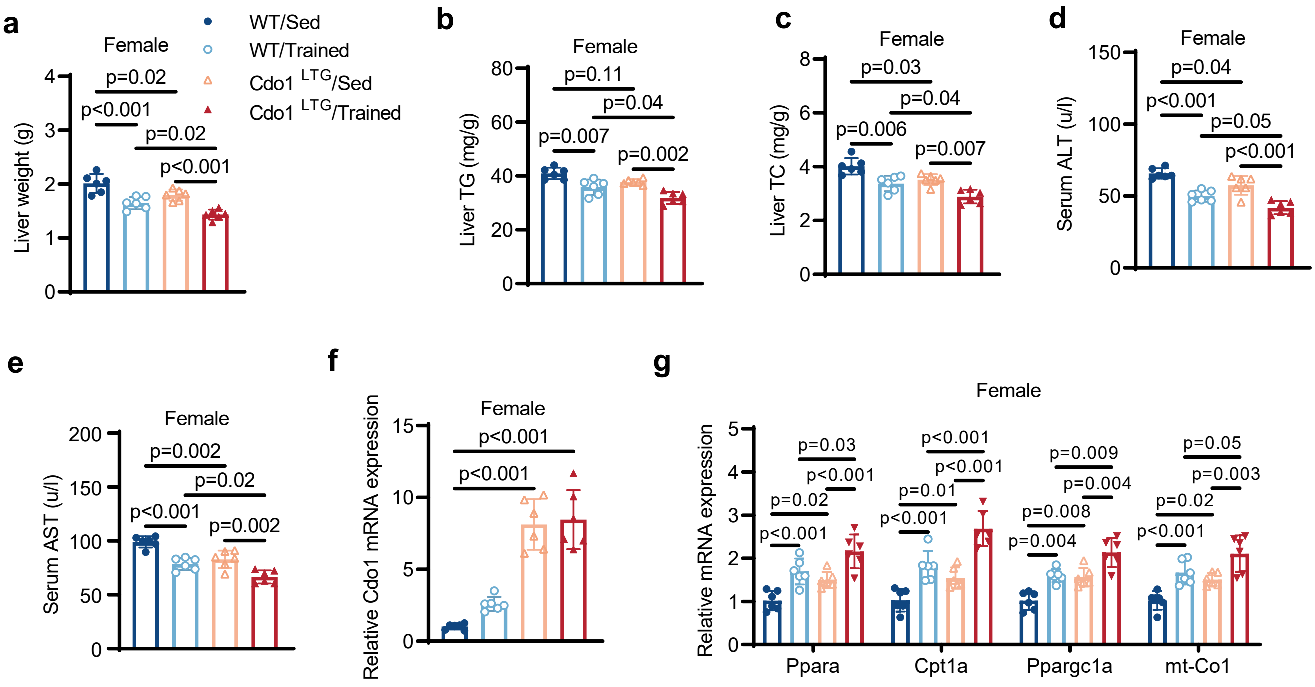


**Supplementary Fig.9 Hepatocyte-specific overexpression of Cdo1 (Cdo1$^{LTG}$) alleviates fatty liver in synergy with exercise in female mice.** 6-week-old WT and Cdo1$^{LTG}$ female mice were fed HFD for 16 weeks, with or without exercise in the last 8 weeks. The intervention program is illustrated of **Supplementary Fig. 7b**. Then mice were sacrificed for analysis (n=6 mice per group). **a** Liver weights in mice. **b**, **c** Triglyceride (TG) and Cholesterol (TC) levels in mice livers, respectively. **d**, **e** Serum alanine aminotransferase (ALT) and serum aspartate aminotransferase (AST) levels in mice, respectively. **f** The mRNA levels of the indicated genes were determined in mice liver. Two-way analysis of variance plus Tukey's post hoc tests were performed in **a-f**. All data show the means ± SD. Source data are provided as a Source Data file.

Supplementary Table 1. Primers used for qPCR.

| | Forward primer (5'-3') | Reverse primer (5'-3') |
|---|---|---|
| **ChIP-qPCR primers** | | |
| β-globin promoter | AAGCCTGATTCCGTAGAGCCACAC | CCCACAGGCAAGAGACAGCAGC |
| Cdo1 promoter | GCCTGAACCCTCCTCT | TCAAACAGCCAAGCAC |
| **RT-qPCR primers** | | |
| CPT1A homo | CGGACGGGATTGACCT | CCACCACCACGATAAGC |
| PGC1A Homo | ACGACGAAGCAGACAAG | AATAGGATTGCGTGCC |
| CDO1 homo | GGTGGTTGGTTGTGGTA | CAGTGGGAGTTGGTATGA |
| PPARA Homo | CCTCGGTGACTTATCCTG | ACTGGCATTTGTTTCTGTT |
| mt-CO1 homo | GACCGCAACCTCAACA | AGCCTGGTAGGATAAGAAT |
| 18S rRNA homo | TTCGAACGTCTGCCCTATCAA | ATGGTAGGCACGGCGACTA |
| mt-Co1 mus | TCTATCAATGGGAGCAGTGTTT | GTAGTCTGAGTAGCGTCGTGGT |
| mt-Nd5 mus | ATAGCCTGGCAGACGAACAA | GGAGATTAGGGGGGGAAAAC |
| Ehhadh mus | AACCCATCACATCAAGCA | CCAACTGAGGCAGCATAG |
| Acadm mus | CGTTCTAACCCAGATCCTA | AAACCTGCTCCTTCACC |
| Cpt1a mus | TCCAAGTATCTGGCAGTCG | AGCCGTCATCAGCAACC |
| Ppargc1a mus | ACAAGACTATTGAGCGAACC | GTGGCTGCCTTGGGTA |
| Ndufs1 mus | GCAGGAGTAGATGATTTGG | AAGGCTTAGAGGTTAGGG |
| Ppara mus | GCTATCCCAGGCTTTGC | CATCCCGTCTTTGTTCATC |
| Srebf1 mus | CTTCTGGAGACATCGCAAAC | GGTAGACAACAGCCGCATC |
| Acaca mus | GCTGATCCTGCGAACC | AACCCAAGAACCACCC |
| Elovl6 mus | AACAAGCGAGCCAAGT | TGCATAAGCCCAGAATT |
| Cdo1 mus | ACCCTGGCGGACCTCAT | ACTCGGCGGGATTGCTC |
| 18s rRNA mus | GGGAGCCTGAGAAACGG | GCTGGCACCAGACTTGC |
| 36b4 mus | AGATTCGGGATATGCTGTTGGC | TCGGGTCCTAGACCAGTGTTC |
| **Primer sequences for genomic DNA** | | |
| Gene hexokinase-2 mus | GCCAGCCTCTCCTGATTTTAGTGT | GGGAACACAAAAGACCTCTTCTG G |
| Gene mtCO1 mus | GCCCCAGATATAGCATTCCC | GTTCATCCTGTTCCTGCTCC |
| Gene mtCO1 homo | GACCGCAACCTCAACA | CGAAGCCTGGTAGGATAA |
| Gene hexokinase-2 homo | GGTCCTGATGCGGTTGG | TCGCCTTTGTTCTCCTTGAT |
