# Supplementary material for: Cdo1-Camkk2-AMPK axis confers the protective effects of exercise against NAFLD in mice
Source: Nat Commun. 2023 Dec 18;14:8391. doi: 10.1038/s41467-023-44242-7 (PMC10728194; doi:10.1038/s41467-023-44242-7)
Supplement: Supplementary file 3 — Reporting Summary [file 41467_2023_44242_MOESM3_ESM.pdf]

nature portfolio

Corresponding author(s): Liang Guo

Last updated by author(s): Oct 16, 2023

Reporting Summary

Nature Portfolio wishes to improve the reproducibility of the work that we publish. This form provides structure for consistency and transparency in reporting. For further information on Nature Portfolio policies, see our Editorial Policies and the Editorial Policy Checklist.

Statistics

For all statistical analyses, confirm that the following items are present in the figure legend, table legend, main text, or Methods section.

n/a	Confirmed	
☐	☒	The exact sample size (n) for each experimental group/condition, given as a discrete number and unit of measurement
☐	☒	A statement on whether measurements were taken from distinct samples or whether the same sample was measured repeatedly
☐	☒	The statistical test(s) used AND whether they are one- or two-sided<br>*Only common tests should be described solely by name; describe more complex techniques in the Methods section.*
☒	☐	A description of all covariates tested
☐	☒	A description of any assumptions or corrections, such as tests of normality and adjustment for multiple comparisons
☐	☒	A full description of the statistical parameters including central tendency (e.g. means) or other basic estimates (e.g. regression coefficient) AND variation (e.g. standard deviation) or associated estimates of uncertainty (e.g. confidence intervals)
☐	☒	For null hypothesis testing, the test statistic (e.g. F, t, r) with confidence intervals, effect sizes, degrees of freedom and P value noted<br>*Give P values as exact values whenever suitable.*
☒	☐	For Bayesian analysis, information on the choice of priors and Markov chain Monte Carlo settings
☒	☐	For hierarchical and complex designs, identification of the appropriate level for tests and full reporting of outcomes
☒	☐	Estimates of effect sizes (e.g. Cohen's d, Pearson's r), indicating how they were calculated

Our web collection on statistics for biologists contains articles on many of the points above.

Software and code

Policy information about availability of computer code

Data collection	1. qPCR: QuantStudio 6 Flex by Thermo Fisher Scientific<br>2. Western blot: Tanon-5200S, BIO-RAD<br>3. RNA-seq: Illumina Hiseq Platform<br>4. Cell oxygen consumption rates: Oxygraph-2k, Oroboros, Innsbruck, Austria<br>5. Immunofluorescence: Leica microscope<br>6. Luciferase reporter assays: CLARIOstar by BMG LABTECH<br>7. GTT and ITT: a glucometer monitor (Roche)<br>8. H&E staining and Oil O staining: Olympus microscope<br>9. FAO level: CLARIOstar by BMG LABTECH<br>10. Transmission electron microscopy: HITACHI HT7800<br>11. Metabolic rate: TSE-system, XYZ, 6M/R, Germany Instruments

Data analysis	1. Western blot: ImageJ (Version 1.53K)<br>2. Figures were constructed using Microsoft Power Point (Version 2304)<br>3. Statistical analysis: Graphpad prism (Version 9), IBM SPSS Statistics 27<br>4. . RNA-seq: Clean reads were aligned to the reference genome using Hisat2 v2.0.5, DESeq2 R package (1.20.0), clusterProfiler R package (3.8.1)

For manuscripts utilizing custom algorithms or software that are central to the research but not yet described in published literature, software must be made available to editors and reviewers. We strongly encourage code deposition in a community repository (e.g. GitHub). See the Nature Portfolio guidelines for submitting code & software for further information.

Data

Policy information about availability of data

All manuscripts must include a data availability statement. This statement should provide the following information, where applicable:

- Accession codes, unique identifiers, or web links for publicly available datasets
- A description of any restrictions on data availability
- For clinical datasets or third party data, please ensure that the statement adheres to our policy

The raw RNA-seq data generated in Figure 3a,3b, 3f and Supplementary Fig.2o have been deposited in the GEO datasets under accession code GSE239729 (https://www.ncbi.nlm.nih.gov/geo/query/acc.cgi?acc=GSE239729). The published microarray data generated from the liver tissue of obese mice (GSE83596, https://www.ncbi.nlm.nih.gov/geo/geo2r/?acc=GSE83596) and RNA-seq data from the livers of patients (GSE126848, https://www.ncbi.nlm.nih.gov/geo/geo2r/?acc=GSE126848) with or without NAFLD in the GEO database were analyzed and shown in Figure 2a and 2b. The data used to generate the main results shown in the main figures and Supplementary figures are available as Source data. Source data are provided with this paper.

Research involving human participants, their data, or biological material

Policy information about studies with human participants or human data. See also policy information about sex, gender (identity/presentation), and sexual orientation and race, ethnicity and racism.

Reporting on sex and gender	N/A
Reporting on race, ethnicity, or other socially relevant groupings	N/A
Population characteristics	N/A
Recruitment	N/A
Ethics oversight	N/A

Note that full information on the approval of the study protocol must also be provided in the manuscript.

Field-specific reporting

Please select the one below that is the best fit for your research. If you are not sure, read the appropriate sections before making your selection.

☒ Life sciences ☐ Behavioural & social sciences ☐ Ecological, evolutionary & environmental sciences

For a reference copy of the document with all sections, see nature.com/documents/nr-reporting-summary-flat.pdf

Life sciences study design

All studies must disclose on these points even when the disclosure is negative.

Sample size	Sample size was determined on experiment feasibility and material availability.
Data exclusions	No data was excluded.
Replication	All data show the means ± standard deviation (SD) of at least three biological replicates with the n indicated in each experiment. All attempts at replication were successful.
Randomization	Cell samples and research animals were randomly assigned to different groups.
Blinding	The treatments provided to mice and cell were not blinded for practical reasons to avoid mix up. To prevent bias, the investigators were blinded to group allocation during data collection and analysis.

Reporting for specific materials, systems and methods

We require information from authors about some types of materials, experimental systems and methods used in many studies. Here, indicate whether each material, system or method listed is relevant to your study. If you are not sure if a list item applies to your research, read the appropriate section before selecting a response.

Materials & experimental systems

n/a	Involved in the study	
☐	☒	Antibodies
☐	☒	Eukaryotic cell lines
☒	☐	Palaeontology and archaeology
☐	☒	Animals and other organisms
☒	☐	Clinical data
☒	☐	Dual use research of concern
☒	☐	Plants

Methods

n/a	Involved in the study	
☒	☐	ChIP-seq
☒	☐	Flow cytometry
☒	☐	MRI-based neuroimaging

Antibodies

Antibodies used

The following commercial antibodies were used:
Anti-Cdo1 (Proteintech, Cat:12509-1-AP, Lot:00057877, dilution 1:1000);
Anti-AMPKα (Proteintech, Cat: 10929-2-AP, Lot: 00115023, dilution 1:1000);
Anti-p-AMPKα (Beyotime, Cat: AA393-1, dilution 1:1000);
Anti-CREB1 (Proteintech, Cat: 12208-1-AP, Lot: 00102107, dilution 1:800);
Anti-p-CREB (Proteintech, Cat: 28792-1-AP, Lot:00102484, dilution 1:800);
Anti-ACC1 (Proteintech, Cat: 67373-1-IG, Lot: 10013129, dilution 1:1000);
Anti-p-ACC1 (Santa Cruz, Cat: sc-271965, Lot: E1922, dilution 1:200);
Anti-Camkk2 (Proteintech, Cat: 11549-1-AP, Lot: 00097881, dilution 1:800);
Anti-p-Camkk2 (Affinity, Cat: AF4487, Lot: 8204594,dilution 1:500);
Anti-LKB1(Santa Cruz, Cat: sc-32245, Lot: G1422,dilution 1:200);
Anti-p-Akt(s473) (CST, Cat: 9272S, Lot:28,dilution 1:1000);
Anti-tAKT (CST, Cat: 4060T, Lot:25, dilution 1:1000);
Anti-HA (Proteintech, Cat: 51064-2-AP, Lot: 00116648,dilution 1:10000)
Anti-Flag (Proteintech, Cat: 66008-4-Ig, Lot:10027647, dilution 1:6000);
Anti-GAPDH (CST, Cat: D16H11, Lot: 8, dilution 1:1000) ;
Anti-HSP90α/β (Santa Cruz, Cat: sc-13119, Lot: Jo722, dilution 1:1000);
Peroxidase affiniPure goat anti-mouse IgG secondary antibody (Jackon, Cat: 111-035-003, Lot: 151083, dilution 1:3000);
peroxidase AffiniPure goat anti-rabbit IgG secondary antibody (Jackon, Cat: 111-035-003, Lot: 153526, dilution 1:3000);
Goat anti-mus IgG/Alex Fluor 555 (Beyotime, Cat: A0460, dilution 1:500);
Goat anti-Ribbit IgG/Alex Fluor 488 (Beyotime, Cat: A0562, dilution 1:500).

Validation

Anti-Cdo1 (Proteintech, Cat:12509-1-AP),
https://www.ptgcn.com/products/CDO1-Antibody-12589-1-AP.htm

Anti-AMPKα (Proteintech, Cat: 10929-2-AP)
https://www.ptgcn.com/products/PRKAA1-Antibody-10929-2-AP.htm

Anti-p-AMPKα (Beyotime, Cat: AA393-1)
https://www.beyotime.com/product/AA393.htm

Anti-CREB1 (Proteintech, Cat: 12208-1-AP),
https://www.ptgcn.com/products/CREB1-Antibody-12208-1-AP.htm

Anti-p-CREB (Proteintech, Cat: 28792-1-AP)
https://www.ptgcn.com/products/Phospho-CREB1-Ser133-Antibody-28792-1-AP.htm

Anti-ACC1 (Proteintech, Cat: 67373-1-IG)
https://www.ptgcn.com/products/ACC-Antibody-67373-1-Ig.htm

Anti-p-ACC1 (Santa Cruz, Cat: sc-271965),
https://www.scbt.com/p/p-accalpha-antibody-f-2?requestFrom=search

Anti-Camkk2 (Proteintech, Cat: 11549-1-AP)
https://www.ptgcn.com/products/CAMKK2-Antibody-11549-1-AP.htm

Anti-p-Camkk2 (Affinity, Cat: AF4487)
https://www.affbiotech.cn/goods-14822-AF4487-Phospho_CaMKK2_Ser511_Antibody.html

Anti-LKB1(Santa Cruz, Cat: sc-32245)

https://www.scbt.com/p/lkb1-antibody-ley-37d-g6?requestFrom=search

Anti-pAkt-473 (CST, Cat: 9272S, Lot:28)
https://www.cellsignal.cn/products/primary-antibodies/phospho-akt-ser473-d9e-xp-rabbit-mab/4060?site-search-type=Products&N=4294956287&Ntt=d9e&fromPage=plp

Anti-Anti-Akt (CST, Cat: 4060T, Lot:25)
https://www.cellsignal.cn/products/primary-antibodies/akt-antibody/9272?site-search-type=Products&N=4294956287&Ntt=9272s&fromPage=plp&_requestid=1502154

Anti-HA (Proteintech, Cat: 51064-2-AP)
https://www.ptgcn.com/products/HA-tag-Antibody-51064-2-AP.htm

Anti-Flag (Proteintech, Cat: 66008-4-Ig)
https://www.ptgcn.com/products/Flag-tag-Antibody-66008-4-Ig.htm

Anti-GAPDH (CST, Cat: D16H11, Lot: 8)
https://www.cellsignal.com/products/primary-antibodies/gapdh-d16h11-xp-rabbit-mab/5174?site-search-type=Products&N=4294956287&Ntt=d16h11&fromPage=plp

Anti-HSP90α/β (Santa Cruz, Cat: sc-13119, Lot: Jo722).
https://www.scbt.com/zh/p/hsp-90alpha-beta-antibody-f-8?requestFrom=search

Goat anti-mouse (Jackon, Cat: 111-035-003, Lot: 151083),
http://www.neobioscience.com/prod_view.aspx?TypeId=236&Id=726611&FId=t3:236:3

Eukaryotic cell lines

Policy information about cell lines and Sex and Gender in Research

Cell line source(s)	HEK293T (American Type Culture Collection, CRL3216, USA), HEK293A (National Collection of Authenticated Cell Cultures, SCSP-5094, China), Hepa1-6 (American Type Culture Collection, CRL1830, USA), HepG2 (National Collection of Authenticated Cell Cultures, SCSP-510, China).
Authentication	The cell lines were authenticated using STR method by the suppliers.
Mycoplasma contamination	All cell lines were tested negative for mycoplasma contamination.
Commonly misidentified lines (See ICLAC register)	No commonly misidentified cell lines were used.

Animals and other research organisms

Policy information about studies involving animals; ARRIVE guidelines recommended for reporting animal research, and Sex and Gender in Research

Laboratory animals	All animal experiments were approved by Shanghai University of Sport Animal Care and Use Committee (No. 102772022DW002). C57BL/6J mice were used for all the experiments. Cdo1 flox/flox mice were generated by CRISPR–Cas9 with loxP sites flanking exon 3, and the genotype was identified via PCR with the following sequences: forward AAGTTTGTGTTTTGTTACCGTGTC, reverse GTCCGTGTGATCATGAATACTGC. Albumin-Cre mice were purchased from the Model Animal Research Center of Nanjing University. Liver specific Cdo1 knockout mice (Cdo1flox/folx/Albumin- Cre+, Cdo1LKO) were generated by crossbreeding Cdo1flox/flox and albumin promoter driven Cre transgenic mice. Liver specific Cdo1 transgenic mice were generated by cross-breeding the mice containing the CMV promoter-driven but stop signal-suppressed 3*Flag-tagged murine Cdo1 expression cassette (CMV-Stop-Cdo1 mice) with Albumin-Cre mice. Albumin promoter-driven expression of Cre was used to remove the stop signal so as to specifically trigger the expression of 3*Flag-tagged Cdo1 in hepatocytes, thereby generating Cdo1 LTG mice. CMV-Stop-Cdo1/Albumin-Cre- mice were used as the controls (wild-type, WT). All animals were housed at 23± 2°C with a humidity of 50% ± 5% in a 12 h light/dark cycle and fed ad libitum with standard mouse feed and water throughout the experiments. Further, 6-week-old mice with different genotypes as described above were fed on HFD (D12492, Research Die) for 16 weeks to cause diet-induced-NAFLD. Chow diet (CD, 1010086, Xietong Shengwu, China) was used as the control diet. Beside HFD-induced NAFLD, mice were in generally good health.
Wild animals	No wild animals were used in this study.
Reporting on sex	We used male and female C57BL6/J mice in our study. In our present work, the functional role of liver Cdo1 in controlling NAFLD and in exercise-mediated alleviation of NAFLD was explored in both male and female mice.<br>For cell experiments, we used HepG2 (male) and Hepa1-6 cells (sex unknown), and primary hepatocytes were isolated from liver tissue of 8-week-old male mice.
Field-collected samples	No field collected samples were used in this study.
Ethics oversight	The procedures related to animal subjects were approved by Shanghai University of Sport Animal Care and Use Committee (No. 102772022DW002).

Note that full information on the approval of the study protocol must also be provided in the manuscript.
