# Supplementary figures and images for: Author Correction: Cdo1-Camkk2-AMPK axis confers the protective effects of exercise against NAFLD in mice
Source: Nat Commun. 2026 Jul 14;17:6197. doi: 10.1038/s41467-026-75556-x (PMC13370004; doi:10.1038/s41467-026-75556-x)

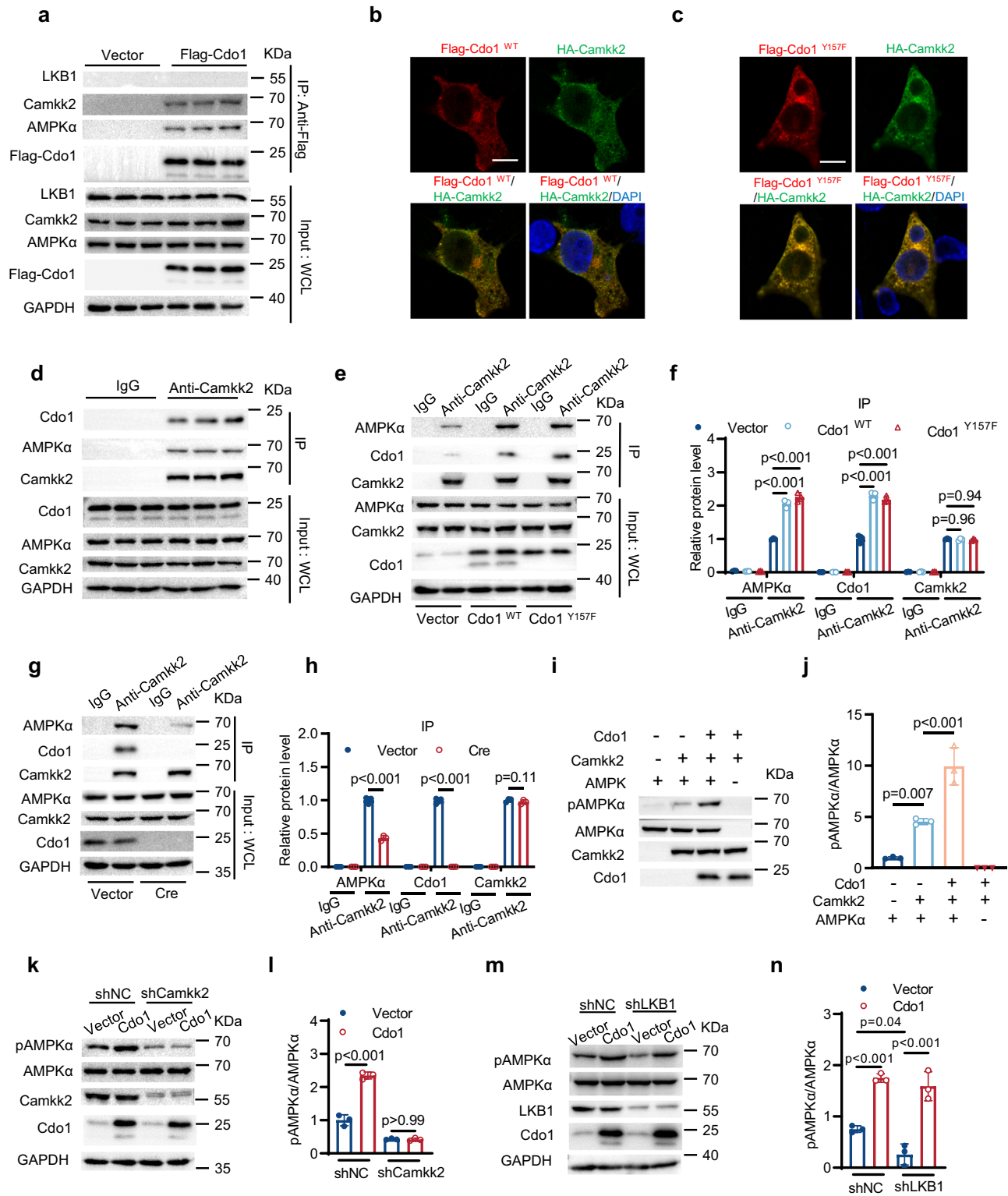

Supplement: Supplementary file 1 — Original Fig. 7 [file 41467_2026_75556_MOESM1_ESM.pdf]
